# High-Temperature Tensile Characteristics of an Al–Zn–Mg–Cu Alloy: Fracture Characteristics and a Physical Mechanism Constitutive Model

**DOI:** 10.3390/ma17112628

**Published:** 2024-05-29

**Authors:** Daoguang He, Yuan Chen, Shibing Chen, Yongcheng Lin, Jiafu Wu

**Affiliations:** 1 School of Mechanical and Electrical Engineering, Central South University, Changsha 410083, China; daoguanghe@csu.edu.cn (D.H.); 233712182@csu.edu.cn (Y.C.); 213711012@csu.edu.cn (S.C.); 2 State Key Laboratory of Precision Manufacturing for Extreme Service Performance, Central South University, Changsha 410083, China

**Keywords:** high-temperature tensile behavior, fracture mechanism, constitutive model, Al–Zn–Mg–Cu alloy

## Abstract

High-temperature tensile tests were developed to explore the flow features of an Al-Zn-Mg-Cu alloy. The fracture characteristics and microstructural evolution mechanisms were thoroughly revealed. The results demonstrated that both intergranular fractures and ductile fractures occurred, which affected the hot tensile fracture mechanism. During high-temperature tensile, the second phase (Al_2_CuMg) at the grain boundaries (GBs) promoted the formation and accumulation of dimples. With the continual progression of high-temperature tensile, the aggregation/coarsening of dimples along GBs appear, aggravating the intergranular fracture. The coalescence and coarsen of dimples are reinforced at higher tensile temperatures or lower strain rates. Considering the impact of microstructural evolution and dimple formation/coarsening on tensile stresses, a physical mechanism constitutive (PMC) equation is herein proposed. According to the validation and analysis, the predictive results were in preferable accordance with the testing data, showing the outstanding reconfiguration capability of the PMC model for high-temperature tensile features in Al–Zn–Mg–Cu alloys.

## 1. Introduction

As a kind of alloy with resistance to corrosion and damage, the Al–Zn–Mg–Cu alloy is essential in a broad range of vital components used in automobiles and aircrafts [1,2,3,4,5,6]. Appropriate atomic elements are often added to obtain improved mechanical properties in Al–Zn–Mg–Cu alloys [7], which results in sophisticated thermal deformation features. Firstly, a multitude of investigations have been conducted to explore the correlation between substructure development (i.e., dislocation emergence/rearrangement [8] via subgrain evolution [9]) and deformation parameters. Moreover, the changing characteristics of dynamic recrystallization exerting impacts upon the deformation features of Al–Zn–Mg–Cu alloys were revealed [10,11]. Furthermore, several studies have investigated the formation/aggregation of dimples affecting high-temperature fracture mechanisms [12]. For instance, Liu et al. [13] found that the dominant form of failure in 7075-aluminum alloys shifted from ductile fracture to brittle fracture with increasing temperature. Zhou et al. [14] exposed that the congregation of dimples around the second phase exhibited a significant influence on the thermal deformation in Al–Zn–Mg–Cu alloys.

In fact, establishing correct constitutive models is critical to exactly numerical simulation and forecasting thermal formation features in alloys [15,16,17,18]. Later, a few constitutive models for reconstituting high-temperature deformation features of alloys were built [19,20,21,22]. Typically, multi-type phenomenological equations [23,24,25,26,27] as well as machine learning models [28,29] are constructed to visualize thermal-flow-forming features in Al–Zn–Mg–Cu alloys and other alloys [30,31,32,33,34]. Additionally, multi-type microstructural changing mechanisms affecting the flow behaviors have been considered, and physical mechanism constitutive (PMC) models have been built [25,35,36,37]. Some PMC models were specially constructed for discerning the interacting impact mechanisms of dislocations, grains, and flow stresses of Al–Zn–Mg–Cu alloys [38,39,40].

Many prior investigations have investigated the high-temperature flow features and microstructural developments in aluminum alloys. Nonetheless, research on the synthesis of fracture features as well as high-temperature-tensile-fracture/forming mechanisms for Al–Zn–Mg–Cu alloys is still lacking. Therefore, this article is devoted to discerning high-temperature tensile performance in an Al–Zn–Mg–Cu alloy. In particular, based on the investigations in refs. [13,28], the dimple emergence/aggregation affecting the high-temperature tensile flow characteristics was explored. Moreover, the formation mechanisms and evolution features of dimples near the second phase were analyzed. The interaction between dimple nucleation/coarsening and the second phases was discussed. Additionally, a physical mechanism constitutive (PMC) model was established for reconstituting the evolution features of the substructure, dimple, and tensile stress in an Al–Zn–Mg–Cu alloy. 

## 2. Experimental Material and Procedure

**Figure 1:**
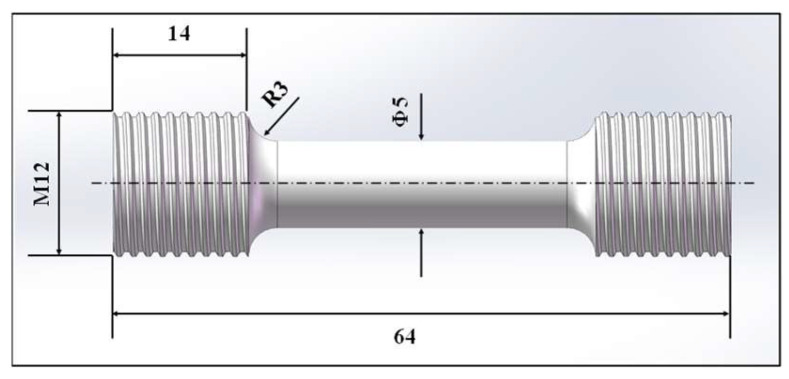
The size of the uniaxial tensile sample (/mm).

An Al–Zn–Mg–Cu (7075 aluminum) alloy was adopted in the current investigation, which was produced by ALG aluminum Inc. (Nanning, China). The chemical composition (wt. %) of the as-received Al–Zn–Mg–Cu alloy was 6.65Zn-1.68Mg-0.25Cu-(bal.) Al. Here, bal stands for balance, which indicates the remaining content of the alloying element. The geometric dimensions of tensile samples are illustrated in Figure 1.

High-temperature tensile experiments were set up on the CMT-5105GL tensile experimental machine. Every sample was initially heated to tensile temperatures ($T_{s}$) using a constant heating rate (15 °C/s), closely followed for 15 min. Subsequently, every specimen became high-temperature under $T_{s}$ ranges of 350–500 °C and strain rates ($\dot{\varepsilon }$) of 0.001–0.1 s^−1^. Since fractures appeared, the formed specimens were cooled to room temperature in the heating furnace. 

**Figure 2:**
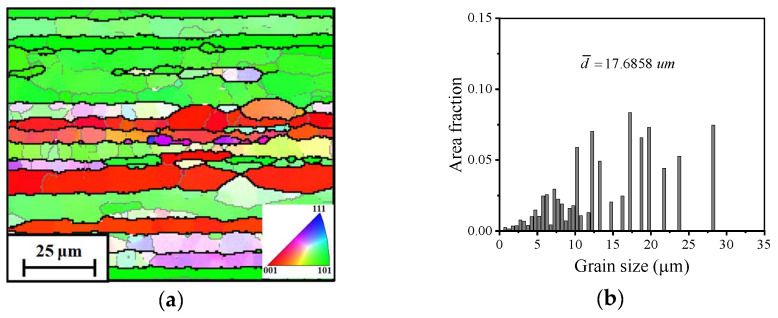
Initial grain characteristics of the Al–Zn–Mg–Cu alloy: (**a**) IPF, (**b**) $\bar{d}$.

The original grain characteristics were discerned by a backscattering electron microscope (EBSD). Before the EBSD observation, the cross-section was obtained from the as-received alloy. Then, these sections were ground with sandpaper and polished with diamond polishing fluid. Furthermore, the polished sections were etched in a solution (20 mL HClO_4_ + 180 mL C_2_H_5_OH). A scanning electron microscope (SEM) was utilized to explore fracture mechanisms. Figure 2 reveals the EBSD result of initial grains, and numerous elongated grains are visible. Accordingly, according to the analysis performed with the Channel 5 software, the mean value of grain size ($\bar{d}$) can be calculated as 17.6858 μm.

## 3. High-Temperature Tensile Characteristics

**Figure 3:**
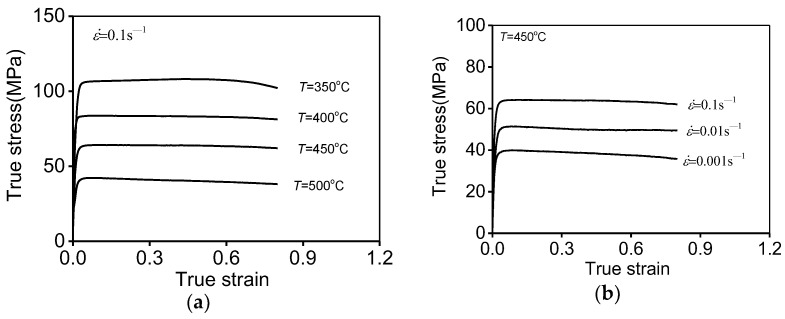
High-temperature tensile features at (**a**) $\dot{\varepsilon }$ = 0.1 s^−1^; (**b**) *T* = 450 °C.

Figure 3 reflects the high-temperature tensile features in the investigative Al–Zn–Mg–Cu alloy.

The evident impacts of tensile parameters on the flow curves can be revealed. The increasing of tensile stresses ($\sigma_{\mathrm{ts}}$) follows the identical tendency as true strain (ε). At the initial small value of ε, the value of σ performs a sharp rising trend for the harden-working (HW) behaviors induced by the growing/interacting of substructures [18]. While the ε constantly increases, the reinforced dynamic-recovery (DRV) mechanism characterized as dislocation rearrangement/annihilation and subgrain development emerges. Synchronously, once the critical strain ($\varepsilon_{c}$) reaches, another softening mechanism (DRX) is activated. Thus, the relative decline in the value of σ can be detected. In the further progression of high-temperature tensile fractures, the development of dimples can occur, which contributes to the notable reduction of $\sigma_{\mathrm{ts}}$.

Additionally, the values of $\sigma_{\mathrm{ts}}$ tend to increase with decreasing $T_{s}$ or ascending $\dot{\varepsilon }$ (Figure 3). This is due to the progression of dislocation cross-slipping/rearrangement, vacancy diffusion, and subgrain development being intensified with decreasing $T_{s}$ or ascending $\dot{\varepsilon }$, inhibiting the DRV behaviors [13]. Moreover, multiple metallurgical characteristics, e.g., subgrain interaction/rotation and the bulging/expansion of GBs [23], can be suppressed at lower $T_{s}$ or higher $\dot{\varepsilon }$ values, which restrains the DRX process [27]. So, the values of $\sigma_{\mathrm{ts}}$ apparently raise with decreasing $T_{s}$ or ascending $\dot{\varepsilon }$. 

## 4. Analysis of Fracture Mechanisms 

**Figure 4:**
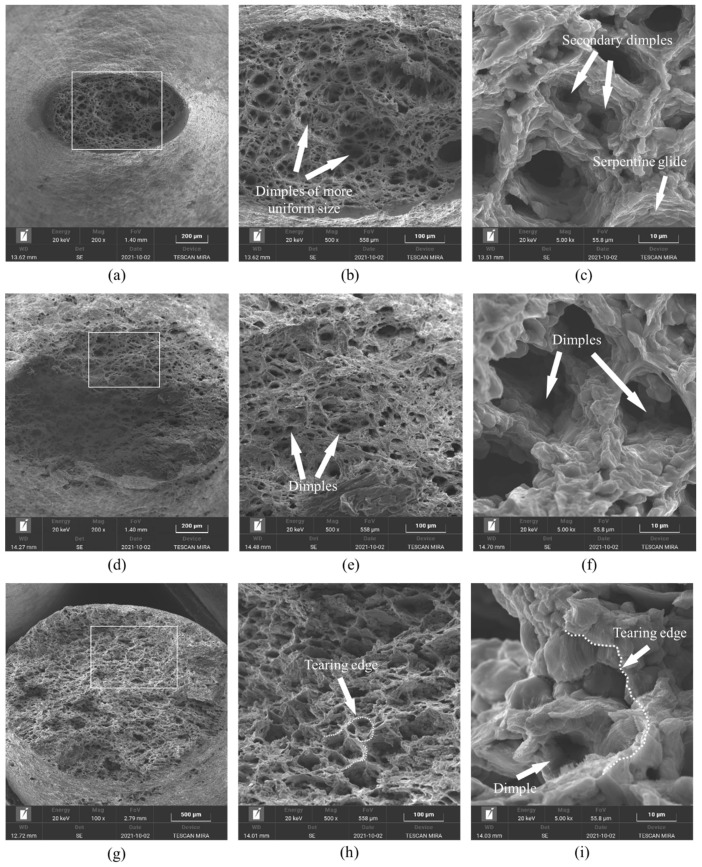
The SEM micrographs at different temperatures of: (**a**–**c**) *T* = 350 °C; (**d**–**f**) *T* = 450 °C; and (**g**–**i**) *T* = 500 °C. Here, the micrographs of (**b**,**c**), (**e**,**f**) and (**h**,**i**) are the high-magnification analysis results of microstructure in the white box region of (**a**), (**d**) and (**g**), respectively.

For the $\dot{\varepsilon }$ at 0.001 $s^{-1}$, the evolution of fracture appearance at different $T_{s}$ is explored in Figure 4. 

Evidently, the local necking feature of the tensile-formed specimens appears, and a few tiny dimples are distributed throughout the fracture surface at 350 °C, demonstrating the occurrence of ductile fracture (Figure 4a). The distribution characteristics of dimples were statistically evaluated using the Image J2 software. Moreover, the evolution of dimples showed a tendency to generate numerous tiny dimples rather than enlarge the anteriority small ones (Figure 4b), which matches the findings in a previous investigation [28]. As revealed in a high-resolution SEM picture (Figure 4c), the serpentine sliding characteristic and some inclusions distributed within dimples can be detected. With the $T_{s}$ increasing to 450 °C (Figure 4d,e), the dimples on the fracture surface became deeper and the coalescence of dimples became obvious. Concurrently, some typical ductile fracture features, i.e., serpentine sliding as well as tenacity nests, can be discovered (Figure 4f). The main aspect of these results is that the vacancy migration, dislocation sliding, and the GB extension were promoted at higher $T_{s}$, which exacerbated the mechanisms of dimple coalescence as well as serpentine slippage. As the $T_{s}$ reached up to 500 °C, massive tiny dimples descended and coalesced to form deeper dimples (Figure 4g,h). Additionally, the tearing behaviors of dimple edges and serpentine gliding tendencies on the interior walls of dimple tended to become distinct (Figure 4i). This is because the DRV progression can also be reinforced at 500 °C [28]. The substructural interaction/annihilation tended to enhance, which reduced the localized concentration. Simultaneously, the conspicuous DRX development activated when the $T_{s}$ of the Al–Zn–Mg–Cu alloy surpassed 400 °C. Thus, promoting the extension rate of DRX GBs encourages the capacity of uniform forming of GBs at 500 °C, which inhibits the generation of tiny dimples. 

**Figure 5:**
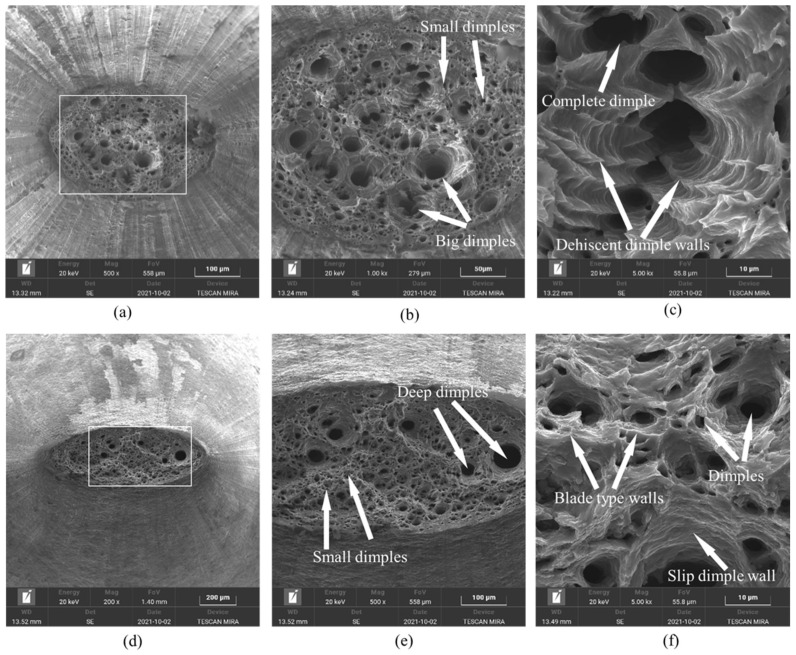
The SEM micrographs at different temperatures of: (**a**–**c**) $\dot{\varepsilon }=0.01\;s^{-1}$ and (**d**–**f**) $\dot{\varepsilon }=0.1\;s^{-1}$. Here, the micrographs of (**b**,**c**) and (**e**,**f**) are the high-magnification analysis results of microstructure in the white box region of (**a**) and (**d**), respectively.

For $T_{s}$ of 400 °C, the evolution of fracture characteristics with $\dot{\varepsilon }$ is explored in Figure 5.

Evidently, the representative local necking feature can be discovered at the $\dot{\varepsilon }$ of 0.01 s^−1^, and massive dimples are distributed in the fracture surface (Figure 5a). Moreover, some regions among dimples showed tearing characteristics owing to the local necking effect, and typical tearing fracture edges appeared (Figure 5b). This is because the differential migration rate on different inner wall regions of dimple induces the appearance of blade-like tearing edges in high-temperature tensile fractures [13]. Besides, visible serpentine gliding features as well as tenacity nests were found (Figure 5c). With the $\dot{\varepsilon }$ increasing to 0.1 s^−1^(Figure 5d,e), the amount of tiny dimples increased, in contrast to that of at 0.01 s^−1^. The aggregation of dimples was inhibited at 0.1 s^−1^, and the tearing features between adjacent dimples were weakened (Figure 5f). This is because the vacancy migration and dislocation rearrangement/annihilation are restrained at higher $\dot{\varepsilon }$, impeding the generation/coalescence of tiny dimples [35]. Additionally, the tendencies of the mobility and tearing on inner walls of dimples become weaken at higher $\dot{\varepsilon }$. 

**Figure 6:**
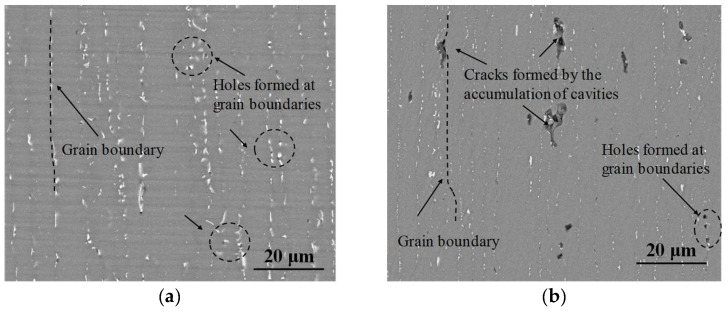
SEM observations of longitudinal section fracture morphology at (**a**) 450 °C/0.1 s^−1^ and (**b**) 500 °C/0.001 s^−1^.

As abovementioned, some conclusions/phases appear as interior dimples. The SEM images for further exploring the interactions between phases and dimples are shown in Figure 6.

Clearly, the precipitation of the second phase exerts a large influence on the formation of dimples/cracks during high-temperature deformation. As unveiled in Figure 6a, massive granular second phases scattered along the GBs at 450 °C/0.1 s^−1^. Concurrently, the generation of tiny dimples around these second phases is clearly visible. Besides, when the tensile parameter was chosen as 500 °C/0.001 s^−1^, the dimples around the second phases represent the coalescence tendency, and cracks can also be detected in Figure 6b. Commonly, the second phase acts as the obstacle for dislocations migrations, which results in high-density dislocations plied along GBs. Then, the superior localized stress concentration appears near the second phases in the GBs, which aggravates the generation of dimples. With the continuous increase in high-temperature tensile stress, dimples undergo coalescence and form cracks. 

**Figure 7:**
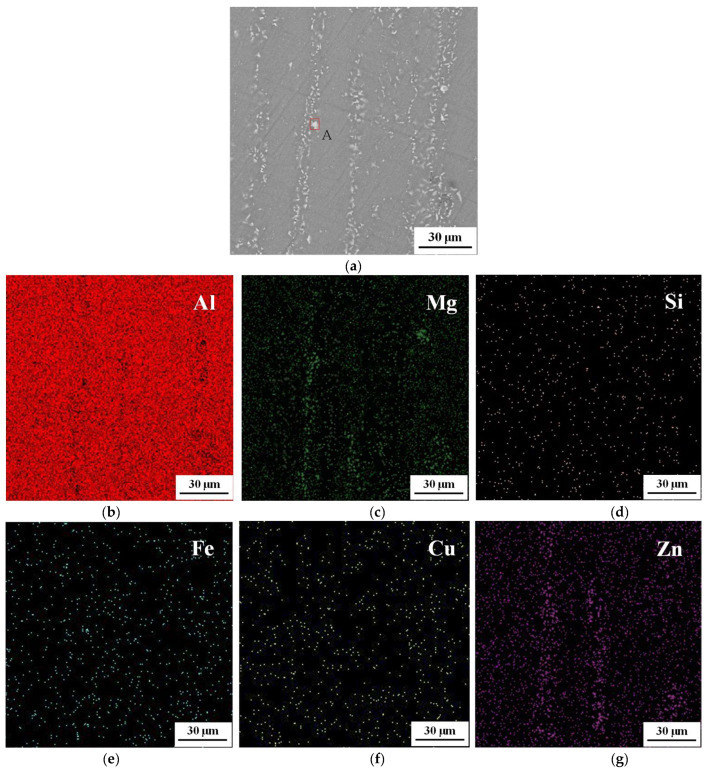
Microstructure maps of (**a**) SEM at 400 °C/0.1 s^−1^. (**b**–**g**) Distribution of elements of Al, Mg, Si, Fe, Cu, and Zn by EDS.

To reveal the composition of the second phases, an analysis of the energy dispersive spectrum (EDS) was performed. Figure 7 shows the morphology and EDS analysis results of the second phases. These second phases were categorized into two main groups: one is the Al_7_Cu_2_Fe phase containing Fe elements, which is resistant to solubilization and conversion. The other is the Al_2_Cu/Al_2_CuMg phase, which can be solvated and transformed through during high-temperature tensile stress.

## 5. The Physical Mechanism Constitutive Model 

### 5.1. Architecture of the Physical Mechanism Constitutive Model

Usually, the variating features of $\sigma_{\mathrm{ts}}$ for Al–Zn–Mg–Cu alloys in high-temperature tensile stress are correlated with various physical mechanisms, e.g., HW (hard working), DRV, and DRX. Correspondingly, the $\sigma_{\mathrm{ts}}$ can be represented as [41]

(1) $$\;\sigma_{\mathrm{ts}}=\sigma_{\mathrm{ys}}+M\alpha \mu b\sqrt{\rho_{i}}-\sigma_{\mathrm{gs}}$$

where the Taylor factor (M) equals 3.06 [20], the burger vector (b) equals 2.86 × 10^−10^ [42], the material coefficient (α) equals 0.15, μ identifies the shear modulus, $\rho_{i}$ identifies the dislocation density, $\sigma_{\mathrm{gs}}$ identifies the stress relevant to grain size evolution, and the yield stress ($\sigma_{\mathrm{ys}}$) is [43]

(2) $$\sigma_{\mathrm{ys}}=A_{y}{\dot{\varepsilon }}^{n_{y}}\exp\left(\frac{-Q_{y}}{RT}\right)$$

where the gas constant (R) identifies 8.314 J/mol·K and $A_{y}$, $Q_{y}$, and $n_{y}$ are material parameters. 

**Figure 8:**
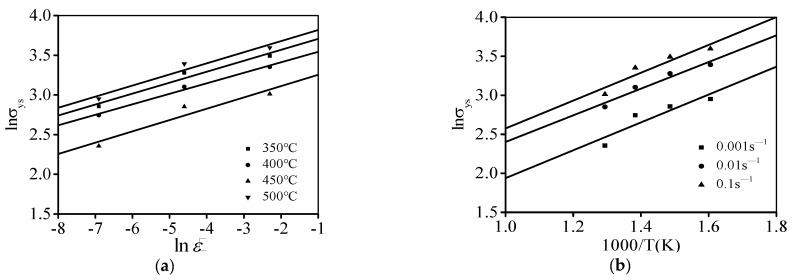
Relationships of (**a**) $\ln\sigma_{\mathrm{ys}}-\ln\dot{\varepsilon }$; (**b**) $\ln\sigma_{\mathrm{ys}}-1000/T$.

Commonly, three material parameters ($A_{y}$, $Q_{y}$, and $n_{y}$) are decided through mathematic relations of $\ln\sigma_{\mathrm{ys}}-\ln\dot{\varepsilon }$ and $\ln\sigma_{\mathrm{ys}}-1/T$ (Figure 8), respectively. Using the linear fitting calculation, the $A_{y}$, $Q_{y}$, and $n_{y}$ are found as 1.8357, 0.1381, and 14,614 J/mol, respectively.

Usually, owing to the impacts of HW, DRV, DRX, and the generation of dimples, the ${\dot{\rho }}_{i}$ is formulated as

(3) $${\dot{\rho }}_{i}={\dot{\rho }}_{i}^{\mathrm{hw}}-{\dot{\rho }}_{i}^{\mathrm{drv}}-{\dot{\rho }}_{i}^{\mathrm{drx}}-{\dot{\rho }}_{i}^{\mathrm{pc}}$$

where ${\dot{\rho }}_{i}$ identifies the evolution rate of $\rho_{i}$; ${\dot{\rho }}_{i}^{\mathrm{hw}}$, ${\dot{\rho }}_{i}^{\mathrm{drv}}$, ${\dot{\rho }}_{i}^{\mathrm{drx}}$ and ${\dot{\rho }}_{i}^{\mathrm{pc}}$ are the evolutive rate of $\rho_{i}$ connected with HW, DRV, DRX, and the dimple evolution mechanisms, respectively.

Normally, the variation of ${\dot{\rho }}_{i}^{\mathrm{hw}}$ and ${\dot{\rho }}_{i}^{\mathrm{drv}}$ is formulated as [42]

(4) $${\dot{\rho }}_{i}^{\mathrm{hw}}=\frac{Mf_{h}\sqrt{\rho_{i}}}{b}\dot{\varepsilon }$$

(5) $${\dot{\rho }}_{i}^{\mathrm{drv}}=f_{v}\rho_{i}\dot{\varepsilon }$$

where the material parameters ($f_{h}\;\mathrm{and}\;f_{v}$) are found by,

(6) $$f_{h}=A_{h}{\dot{\varepsilon }}^{n_{h}}\exp{\left(\frac{Q_{h}}{RT}\right)}^{n_{h1}}$$

(7) $$f_{v}=A_{v}{\dot{\varepsilon }}^{n_{v}}\exp{\left(\frac{Q_{v}}{RT}\right)}^{n_{v1}}$$

where $A_{h},Q_{h},n_{h},n_{h1},A_{v},Q_{v},n_{v}\mathrm{and}\;n_{v1}$ are the material parameters.

Meanwhile, the variation of ${\dot{\rho }}_{i}^{\mathrm{drx}}$ is found as [2]

(8) $${\dot{\rho }}_{i}^{\mathrm{drx}}=f_{x}\frac{\rho_{i}-\rho_{i0}}{1-X_{f}}\dot{S}$$

(9) $$f_{x}=A_{x}{\dot{\varepsilon }}^{n_{x}}\exp{\left(\frac{Q_{x}}{RT}\right)}^{n_{x1}}$$

where $X_{f}$ identifies the DRX fraction; $A_{x},Q_{x},n_{x}\mathrm{and}\;n_{x1}$ are material parameters; $\rho_{i0}=1\times 10^{28}m^{-2}$ is the original value of $\rho_{i}$; and the gradient of $X_{f}$($\dot{S}$) is found as

(10) $$\dot{S}=\frac{\partial X_{f}}{\partial t}=\frac{\partial X_{f}}{\partial \varepsilon }\times \frac{\partial \varepsilon }{\partial t}={\dot{X}}_{f}\cdot \dot{\varepsilon }$$

Commonly, the $X_{f}$ can be confirmed by [40]

(11) $$X_{f}=1-\exp[a{\left(\frac{\varepsilon -\varepsilon_{c}}{\varepsilon_{c}}\right)}^{f_{d}}]\;(\varepsilon \geq \varepsilon_{c})$$

(12) $$\varepsilon_{c}=0.85\varepsilon_{p}$$

(13) $$f_{d}=A_{d}{\dot{\varepsilon }}^{n_{d}}\exp\left(\frac{Q_{d}}{RT}\right)$$

where the $\varepsilon_{c}$ and $\varepsilon_{p}$ identify the critical strain as well as peak strain, respectively; and $a,A_{d},Q_{d}\;\mathrm{and}\;n_{d}$ identify material parameters. 

For alloys in high-temperature tensile stress, the variation of $\rho_{i}$ relating to the dimple development (${\dot{\rho }}_{i}^{\mathrm{pc}}$) is confirmed by [20]

(14) $${\dot{\rho }}_{i}^{\mathrm{pc}}=f_{p}\rho_{i}\dot{\varepsilon }$$

(15) $$f_{p}=A_{p}\varepsilon^{n_{p}}{\dot{\varepsilon }}^{n_{p1}}\exp\left(\frac{Q_{p}}{\mathrm{RT}}\right)$$

where the $A_{p},Q_{p},n_{p},\mathrm{and}\;n_{p1}$ are material parameters and $Q_{p}$ is the dimple activation energy. 

Additionally, the $\sigma_{\mathrm{gs}}$ can be confirmed by [2]

(16) $$\sigma_{\mathrm{gs}}=f_{g}X_{f}d^{-1/2}$$

where d is the grain size and $f_{g}$ is the material coefficient. The values of these are defined as [44]

(17) $$f_{g}=A_{g}{\dot{\varepsilon }}^{n_{g}}\exp\left(\frac{Q_{g}}{RT}\right)$$

(18) $$\dot{d}=(d_{drx}-\frac{4}{3}d_{0}{\left(1-X_{f}\right)}^{1/3}){\dot{X}}_{f}$$

where $d_{0}$ is the original grain size; $\dot{d}$ is the the variating rate of d; and $d_{drx}$ is the the DRX grain size, the value of which is defined as [45]

(19) $$d_{\mathrm{drx}}=A_{\mathrm{drx}}{\dot{\varepsilon }}^{n_{drx}}\exp\left(\frac{-Q_{drx}}{RT}\right)$$

### 5.2. Determination of the Material Parameters of the Physical Mechanism Constitutive Model

To ascertain the material parameters in Equations (1)–(19), the multi-objective optimizations functions are chosen as

(20) $$\min{\sum \left(\sigma_{\mathrm{ts}}^{p}-\sigma_{\mathrm{ts}}\right)}^{2}$$

(21) $$\min{\sum \left(X_{f}^{p}-X_{f}\right)}^{2}$$

(22) $$\min{\sum \left(d^{p}-d\right)}^{2}$$

where $\sigma_{\mathrm{ts}}^{\;p}$, $X_{f}^{\;p}$ and $d^{p}$ are the forecasting values of $\sigma_{\mathrm{ts}}$, $X_{f}$ and d, respectively. 

For identifying the materials parameters of the physical mechanism model, an owl optimization algorithm was adopted [2]. The initial values of the material parameters of $A_{h},Q_{h},n_{h},n_{h1},A_{v},Q_{v},n_{v},\;n_{v1}$, $A_{x},Q_{x},n_{x},\;\mathrm{and}\;n_{x1}$ were chosen as 450,000.0, 140.0, −0.0009, 0.2000, 200.0, −3000.0, 0.08, 0.00004, −28,000, −120.0, −0.8, and 0.55, respectively. Meanwhile, the initial values of the material parameters of $A_{d}$, $Q_{d}$, $n_{d}$, $A_{g}$, $Q_{g}$, $n_{g}$, $A_{drx}$, $Q_{drx}$, $n_{drx}$, $A_{p}$, $Q_{p}$, $n_{p}$, and $n_{p1}$ are 3.0, −6000, −0.01, 1.5 × 10^−4^, 20.0, −0.7, 10,000, 4000, 0.007, 400, 30,000, 0.4, and 20.0, respectively. During the current optimized processing of material parameters, the values of parameters obtained in the former optimized process were chosen as the initial values of material parameters in the next optimized process. For each optimized process of material parameters, the confidence intervals for parameters were set to the positive and negative 200% of the former optimized material parameters. Accordingly, the optimized material parameters are itemized in Table 1. 

### 5.3. Validation and Analysis

**Figure 9:**
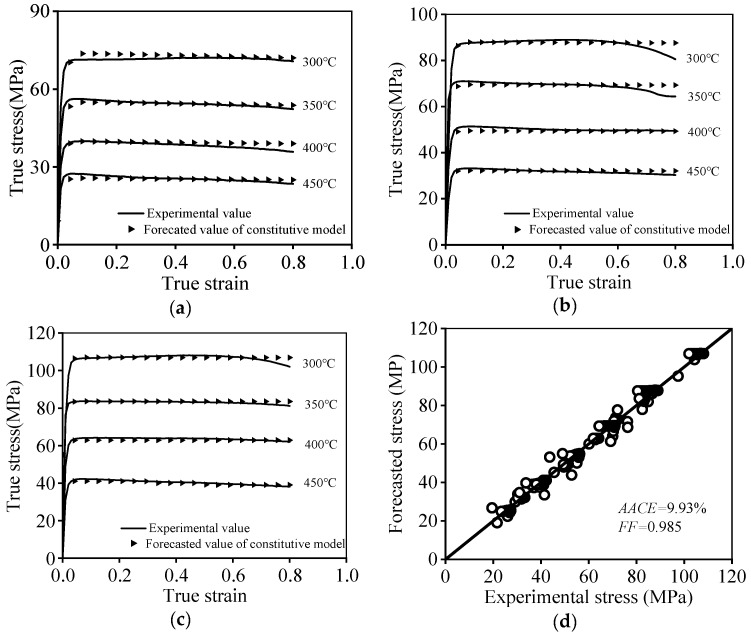
Comparisons of the flow stress at (**a**) $\dot{\varepsilon }$ = 0.001 s^−1^, (**b**) $\dot{\varepsilon }$ = 0.01 s^−1^, (**c**) $\dot{\varepsilon }$ = 0.1 s^−1^, and (**d**) correlation coefficient.

By means of the above-optimized material parameters, the contrastive analyzed results of predictive $\sigma_{\mathrm{ts}}$ and testing ones are shown in Figure 9.

Apparently, the favorable consistency between predictive $\sigma_{\mathrm{ts}}$ and experimental ones can be noticed. Correspondingly, two evaluation error indexes, i.e., fitted factor (FF) as well as average absolute correlation error (AACE) are gained for further assessing the effect of the PM model. The FF and AACE can be acquired by

(23) $$AACE\left(\%\right)=\frac{1}{N}\sum_{i=1}^{N}\left|\frac{{\left(\sigma_{\mathrm{ts}}\right)}_{i}-{\left(\sigma_{\mathrm{ts}}^{\;p}\right)}_{i}}{{\left(\sigma_{\mathrm{ts}}\right)}_{i}}\right|$$

(24) $$FF=\frac{\sum_{i=1}^{N}\left({\left(\sigma_{\mathrm{ts}}\right)}_{i}-{\bar{\sigma }}_{\mathrm{ts}}^{\;p}\right)\left({\left(\sigma_{\mathrm{ts}}^{\;p}\right)}_{i}-{\bar{\sigma }}_{\mathrm{ts}}\right)}{\sqrt{\sum_{i=1}^{N}{\left({\left(\sigma_{\mathrm{ts}}\right)}_{i}-{\bar{\sigma }}_{\mathrm{ts}}\right)}^{2}\sum_{i=1}^{N}{\left({\left(\sigma_{\mathrm{ts}}^{\;p}\right)}_{i}-{\bar{\sigma }}_{\mathrm{ts}}^{\;p}\right)}^{2}}}$$

where ${\bar{\sigma }}_{\mathrm{ts}}^{\;p}$ and ${\bar{\sigma }}_{\mathrm{ts}}$ represent the average values of $\sigma_{\mathrm{ts}}^{p}$ and $\sigma_{\mathrm{ts}}$, respectively. 

According to the calculation analysis, the FF and AACE were determined to be 0.985 and 9.93% (Figure 9d), respectively. These results further demonstrate that the PM model can finely catch the changing features of tensile stress with various structural variating mechanisms, i.e., substructural development and dimple evolution. 

## 6. Conclusions

The fracture morphology/mechanisms and tensile stress of an Al–Zn–Mg–Cu alloy were herein explored. The results are listed as follows.

1. For the Al–Zn–Mg–Cu alloy during high-temperature tensile stress, the ductile fracture, as well as intergranular fracture, mainly contribute to the fracture behavior;
2. The changes of fracture morphology/mechanisms in Al–Zn–Mg–Cu alloys are noticeably affected by high-temperature tensile parameters. The formation/multiplication of dimples is strengthened with reducing $T_{s}$ or increase of $\dot{\varepsilon }$, while the conglomeration/coalescence of dimples becomes weakened;
3. The promoted physical mechanism constitutive (PMC) model, as determined according to the impacts of substructure development and dimple evolution, is proposed. The promoted PMC model enjoys a relatively high value of FF (0.985) and a low value of AACE (9.33%), proving that this model can exactly achieve the reconstitution of high-temperature tensile features.

## Figures and Tables

**Table 1:** Optimal results of material parameters of the physical mechanism model.

Material Parameter	Value	Material Parameter	Value
$A_{h}$	545,002	$Q_{d}$	−6577
$Q_{h}$	100.58	$n_{d}$	−0.008
$n_{h}$	−0.00001	$A_{g}$	1 × 10^−4^
$n_{h1}$	0.21222	$Q_{g}$	24
$A_{v}$	198	$n_{g}$	−0.653
$Q_{v}$	−3859.5	$A_{drx}$	12,173.87
$n_{v}$	0.06	$Q_{drx}$	5505
$n_{v1}$	5.68 × 10^−5^	$n_{drx}$	0.0197
$A_{x}$	−30,000	$A_{p}$	500
$Q_{x}$	−118.2	$Q_{p}$	23,300
$n_{x}$	−0.67	$n_{p}$	0.2333
$n_{x1}$	0.48	$n_{p1}$	24.99
$A_{d}$	2.5		

## Data Availability

The original contributions presented in the study are included in the article, further inquiries can be directed to the corresponding authors.
